# Transmission of HIV and HCV within Former Soviet Union Countries

**DOI:** 10.1155/2020/9701920

**Published:** 2020-07-15

**Authors:** Lazzat Aibekova, Aizada Bexeitova, Arailym Aldabergenova, Gonzalo Hortelano, Zhangwen Ge, Feng Yi, Yiming Shao, Jack DeHovitz, Sten H. Vermund, Syed Ali

**Affiliations:** ^1^Department of Biology, School of Science and Humanities, Nazarbayev University, Astana, Kazakhstan; ^2^State Key Laboratory for Infectious Disease Prevention and Control, National Center for AIDS/STD Control and Prevention, Chinese Center for Disease Control and Prevention, Beijing, China; ^3^Department of Medicine, SUNY Downstate Medical Center, Brooklyn, NY, USA; ^4^Yale School of Public Health, New Haven, CT, USA; ^5^Department of Biomedical Sciences, Nazarbayev School of Medicine, Nazarbayev University, Astana, Kazakhstan

## Abstract

**Background:**

Following the collapse of the Union of Soviet Socialist Republic (USSR) in 1991, trans-border mobility increased within the former Soviet Union (FSU) countries. In addition, drug-trafficking and injection drug use began to rise, leading to the propagation and transmission of blood-borne infections within and across the FSU countries. To examine the transmission of blood-borne infections within this region, we analyzed the phylogenetic relationship of publically available sequences of two blood-borne viruses, hepatitis C virus (HCV) and human immunodeficiency virus (HIV), from FSU countries.

**Methods:**

We analysed 614 and 295 NS5B sequences from HCV genotypes 1b and 3a, respectively, from 9 FSU countries. From 13 FSU countries, we analysed 347 HIV *gag* and 1282 HIV *env* sequences. To examine transmission networks and the origins of infection, respectively, phylogenetic and Bayesian analyses were performed.

**Results:**

Our analysis shows intermixing of HCV and HIV sequences, suggesting transmission of these viruses both within and across FSU countries. We show involvement of three major populations in transmission: injection drug user, heterosexual, and trans-border migrants.

**Conclusion:**

This study highlights the need to focus harm reduction efforts toward controlling transmission of blood-borne infections among the abovementioned high-risk populations in the FSU countries.

## 1. Introduction

Following the collapse of the Union of Soviet Socialist Republics (USSR) in 1991, the ensuing economic crisis led to poverty and unemployment in the former Soviet republics. Existing social and cultural ties among the former Soviet Union (FSU) countries and visa-free travel across borders facilitated massive movement of migrants in search of employment [1]. High migration rates in the setting of economic destabilization were accompanied by increased rates of injected drug use, facilitating the transmission of blood-borne viruses such as human immunodeficiency virus (HIV) and hepatitis C virus (HCV) in the region [2, 3]. Due to their geographic location along drug-trafficking routes from Afghanistan, the main hub of opium production and supply for Russia and Europe, there has been increased trafficking and use of injectable drugs in Central Asia [4]. According to the World Health Organization (WHO), in Eastern Europe, 6.8 million people were estimated in 2015 to be positive for antibodies to HCV (3.3% prevalence) and 4.7 million people were living with chronic HCV (2.3% prevalence; 69% viremia rate), while in Central Asia, these figures were 4.5 million (5.4% prevalence) and 1.9 million (2.3% prevalence; 43% viremia rate) people [5]. The number of people living with HIV in Eastern European and Central Asian (EECA) countries, representing the only region in the world with rising HIV incidence, reached 1.6 million by 2016 [6].

The objective of this study was to investigate the epidemiology of blood-borne viruses, namely, HIV and HCV, among FSU countries. Using viral sequences from public databases, we have performed phylogenetic analysis to assess common routes of transmission of these two viruses within FSU countries.

## 2. Methods

### 2.1. Selection and Downloading of Sequences

For HCV, a 234 bp fragment of NS5B gene (corresponding to H77 8322–8555 nt) was studied. We downloaded 614 sequences for genotype 1b and 295 for genotype 3a from the Los Alamos HCV database (http://www.hcv.lanl.gov). Sequences from 9 FSU countries, namely, Russia, Uzbekistan, Tajikistan, Azerbaijan, Belarus, Lithuania, Latvia, Estonia, and Georgia, were used for this analysis. Sequence retrieval and length decisions were based on selecting the most represented gene fragment (and genotype sequences) available for most FSU countries in the said database (Supplementary Figure 1). Genotypes for all the sequences were ascertained using the Oxford HCV Automated Subtyping Tool 2.0 (http://www.bioafrica.net/rega-genotype/html/subtypinghcv.html). For constructing phylogenetic tress, 10 known genotype sequences were used as reference.

For HIV, subtype A *gag* and *env* sequences from Los Alamos HIV Sequence Database (http://www.hiv.lanl.gov) were downloaded. Available sequences from 13 FSU countries, namely, Armenia, Azerbaijan, Belarus, Estonia, Georgia, Kazakhstan, Kyrgyzstan, Latvia, Lithuania, Moldova, Russia, Ukraine, and Uzbekistan, were retrieved (Supplementary Figure 1). Recombinant and duplicate sequences were identified using, respectively, *Recombinant HIV-1 Drawing Tool v2.1.0* and *ElimDupes* (http://www.hiv.lanl.gov), and eliminated. After alignment and trimming, 371 bp long, 334 *gag* sequences (HXB2: 859–1230 nt), and 257 bp long, 1282 *env* sequences (HXB2: 7071–7336 nt) were used for this analysis. To root the tree, 12 reference sequences of nonsubtype A and group M were used from Los Alamos HIV Sequence Database (http://www.hiv.lanl.gov). To construct the tree with risk group information, data from the HIV Los Alamos database were used.

### 2.2. Phylogenetic Analysis

*SeaView v4.6.1* was used to align sequences using the ClustalO algorithm, and the alignments were manually edited using *MEGA v6.06*. The phylogenetic trees were constructed using maximum likelihood method with 100 replicates bootstrap evaluation under the Tamura-Nei model as nucleotide substitution model, including a uniform rate of heterogeneity among sites, as implemented in *MEGAv6.06.* The final tree was refined and visualized using Figtree 1.4.2 software (http://tree.bio.ed.ac.uk/software/). The clusters were picked based on the predominance of sequences from a particular country or risk group.

### 2.3. Bayesian Analysis

To investigate temporal signal in the data set, we used the maximum-likelihood tree for the analysis of correlation between root-to-tip genetic distance and year of sampling in the program *TempEst v1.5.* A Bayesian phylogenetic approach [7] was used for joint estimation of the ages of each of clusters and the demographic history of all of the strains. This was done by analyzing these sequences using the general time reversible (GTR) model [8] plus a gamma distribution among site rate heterogeneities [9] on the basis of the standard Akaike information criterion in a hierarchical likelihood ratio test in our study. Bayesian Markov chain Monte Carlo (MCMC) analyses were performed with the selected nucleotide substitution model using an uncorrelated lognormal relaxed-clock model [10] as implemented in *BEAST, version 1.8.4.* The analyses were performed using a Bayesian SkyGrid coalescent tree prior [11, 12] in order to investigate the demographic histories of HCV or HIV and the degree to which dating estimates are affected by the demographic model chosen. Sequences for which sampling year information was unavailable were excluded from the analysis.

To ensure convergence of parameter estimates, the MCMC was run for 100 million steps and sampled every 10000 steps. The resulting MCMC samples under different demographic models were used to estimate the rates of evolution and tMRCAs with the first 10% removed as burn-in. Convergence of parameters and sufficient sampling were assessed by calculating the effective sample size (ESS) using TRACER v1.6 (http://beast.bio.ed.ac.uk/software/tracer). A final maximum clade credibility (MCC) tree was constructed from the posterior tree distribution using the program Tree Annotator (http://beast.bio.ed.ac.uk) and visualized in *FigTree.*

## 3. Results

### 3.1. HCV

**Figure 1:**
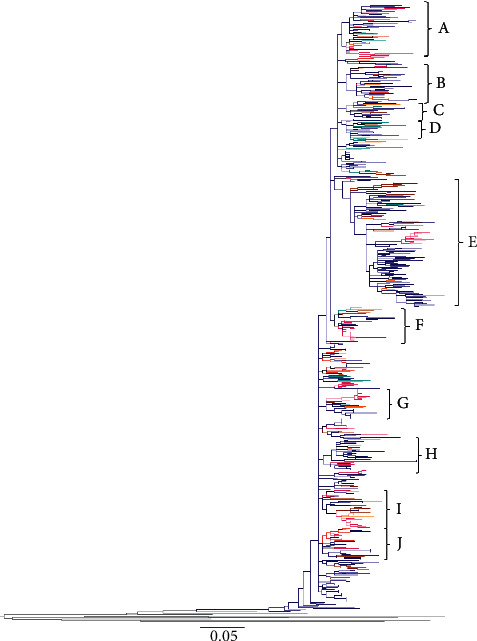
Phylogenetic relationship of HCV genotype 1b NS5B sequences from FSU countries: For the construction of the phylogenetic tree, a stretch of HCV sequence corresponding to H77 nucleotide 8322–8555 was used. The sequences are included from Azerbaijan, Georgia, Estonia, Lithuania, Russia, Tajikistan, and Uzbekistan, represented by, respectively, orange, purple, pink, brown, dark blue, coral, and turquoise branches. The branches coded grey represent 10 outgroup reference sequences that were used to root the tree.

**Figure 2:**
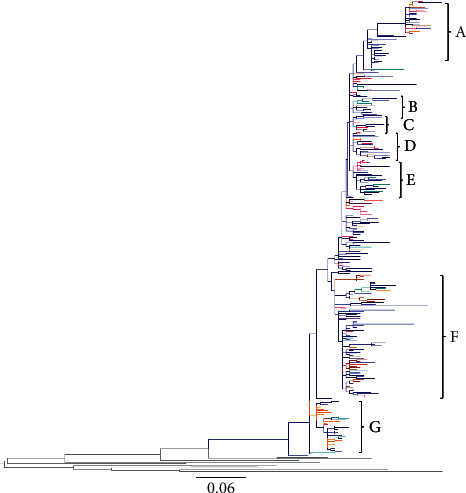
Phylogenetic relationship of HCV genotype 3a NS5B sequences from FSU countries: For the construction of the phylogenetic tree, a stretch of HCV sequence corresponding to H77 nucleotide 8322–8555 was used. The sequences are included from Azerbaijan, Georgia, Estonia, Lithuania, Russia, Tajikistan, and Uzbekistan. The color key is the same as for Figure 1.

The maximum likelihood trees generated using genotype 1b or 3a NS5B sequences from 7 countries revealed phylogenetic links of Russian clusters with sequences from other FSU countries. The sequences analysed were deposited during 1995–2015 (Tables 1 and 2). Overall, 10 clusters were picked from HCV 1b NS5B tree based on the predominance of sequences from a particular country or risk group (Table 1 and Figure 1): cluster A (53 sequences, representing 5 countries), cluster B (37 sequences, representing 7 countries), cluster C (18 sequences, representing 4 countries), cluster D (23 sequences, representing 6 countries), cluster E (133 sequences, representing 6 countries), cluster F (33 sequences, representing 6 countries), cluster G (30 sequences, representing 5 countries), cluster H (31 sequences, representing 4 countries), cluster I (39 sequences, representing 6 countries), and cluster J (32 sequences, representing 5 countries). Genotype 1b tree showed intermixing of Russian sequences mainly with those from Estonia (clusters A, B, and D–J), Lithuania (clusters B-J), Azerbaijan (clusters A-E, G, and I), Uzbekistan (clusters A-G and I), and Tajikistan (clusters A, B, D–F, and H–J) (Figure 1, and Table 1). Seven clusters were picked from HCV 3a NS5B tree based on the predominance of sequences from a particular country or risk group (Table 2 and Figure 2): cluster A (44 sequences, representing 6 countries), cluster B (11 sequences, representing 3 countries), cluster C (11 sequences, representing 4 countries), cluster D (18 sequences, representing 3 countries), cluster E (23 sequences, representing 3 countries), cluster F (80 sequences, representing 6 countries), and cluster G (33 sequences, representing 4 countries). Genotype 3a tree showed a similar trend with an intermixing of HCV sequences from Russia mainly with those from Estonia (clusters A and C–G), Uzbekistan (clusters A, C, and E–G), and Azerbaijan (clusters A, B, D, F, and G) (Figure 2 and Table 2).

### 3.2. HIV

**Figure 3:**
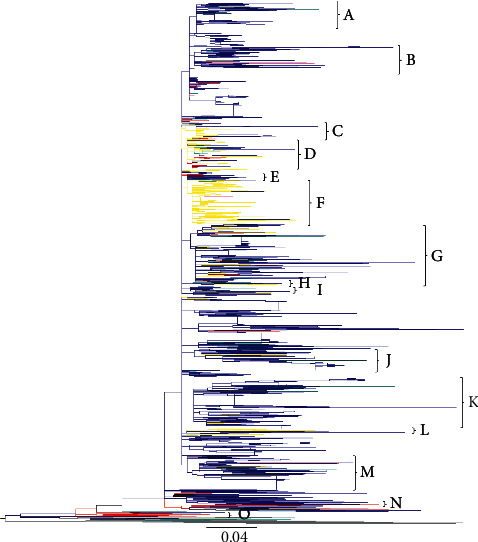
Phylogenetic relationship of HIV subtype A *env* gene sequences from FSU countries: For the construction of the phylogenetic tress, a stretch of HIV sequence corresponding to HBX2 nucleotide 7071–7336 was used. The sequences are included from Armenia, Belarus, Georgia, Estonia, Kyrgyzstan, Latvia, Moldova, Russia, Ukraine and Uzbekistan represented by, respectively, teal, green, purple, pink, neon, yellow, olive, dark blue, red, and turquoise branches. The branches coded grey represent 12 outgroup reference sequences that were used to root the tree.

A phylogenetic tree was constructed with *env* sequences from 10 counties, spanning years 1993–2015, as follows: Armenia (2009), Belarus (1997–2015), Georgia (1997–2013), Estonia (2001–2005), Kyrgyzstan (2009), Latvia (1998–2005), Moldova (1997), Russia (1994–2015), Ukraine (1993–2012), and Uzbekistan (1999–2002). In addition, 12 nonsubtype A references were retrieved from the database and were used as outliers to root the phylogenetic tree. Fifteen clusters were picked from the tree for further analysis (Table 3 and Figure 3): cluster A (76 sequences, representing 5 countries), cluster B (53 sequences, representing 4 countries), cluster C (25 sequences, representing 2 countries), cluster D (83 sequences, representing 6 countries), cluster E (13 sequences, representing 3 countries), cluster F (111 sequences, representing 4 countries), cluster G (143 sequences, representing 5 countries), cluster H (8 sequences, representing 4 countries), cluster I (17 sequences, representing 5 countries), cluster J (55 sequences, representing 4 countries), cluster K (130 sequences, representing 4 countries), cluster L (16 sequences, representing 2 countries), cluster M (84 sequences, representing 5 countries), cluster N (16 sequences, representing 3 countries), and cluster O (6 sequences, representing 1 countries). More than half of the sequences used to construct the tree were from Russia; therefore, the results show a predominance of Russian branches in most clusters (Figure 3 and Table 3). Interestingly, sequences from Russia, Uzbekistan, and Latvia were intermixed in almost all the clusters, whereas sequences from Armenia were also represented prominently in most clusters (Figure 3 and Table 3).

**Figure 4:**
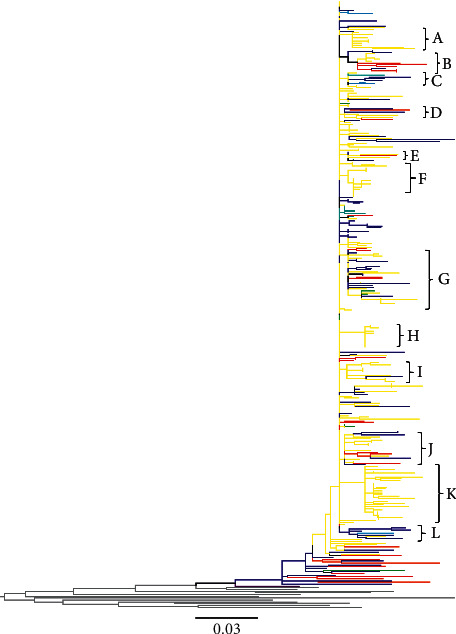
Phylogenetic relationship of HIV subtype A *gag* gene sequences from FSU countries: For the construction of the phylogenetic tress, a stretch of HIV sequence corresponding to HBX2 nucleotide 859–1230 was used. The sequences are included from Belarus, Georgia, Kazakhstan, Latvia, Russia, Ukraine, and Uzbekistan. Color key is the same as for Figure 3.

The second tree was constructed with HIV *gag* sequences from 8 FSU countries spanning the years 1997–2013 as follows: Belarus (1997–2013), Georgia 1999 (1999), Kazakhstan (2002), Latvia (1998–2005), Russia (1997–2011), Ukraine (2000–2012), and Uzbekistan (2002). The majority of *gag* sequences were from Russia and Latvia, whereas only one sequence was from Georgia. Twelve clusters were picked from the tree for the further analysis (Table 4 and Figure 4): cluster A (10 sequences, representing 1 countries), cluster B (13 sequences, representing 3 countries), cluster C (9 sequences, representing 4 countries), cluster D (7 sequences, representing 3 countries), cluster E (6 sequences, representing 4 countries), cluster F (17 sequences, representing 1 countries), cluster G (33 sequences, representing 4 countries), cluster H (13 sequences, representing 1 countries), cluster I (12 sequences, representing 2 countries), cluster J (20 sequences, representing 3 countries), cluster K (31 sequences, representing 1 countries), and cluster L (7 sequences, representing 3 countries). This tree clearly showed intermixing of Russian strains with Latvia (Figure 4). In agreement with the observations from the HIV *env* tree (Figure 3), in this tree as well, sequences from Russia and Latvia were intermixed in almost all the clusters throughout the tree (Figure 4 and Table 4). Additionally, as was observed in the *env* tree, the ancestral sequences, representing the earliest infections, in this tree as well originated from Ukraine, with some intermixing of sequences from Russia (Figures 3 and 4). Both in the *env* (cluster F) and *gag* (clusters A, F, H and K) trees, monophyletic clusters of Latvian sequences originating from Russian sequences were observed (Figures 3 and 4), possibly indicating epidemics in Latvia that resulted from migrant-associated transmissions from Russia.

### 3.3. Risk Group Analysis

**Figure 5:**
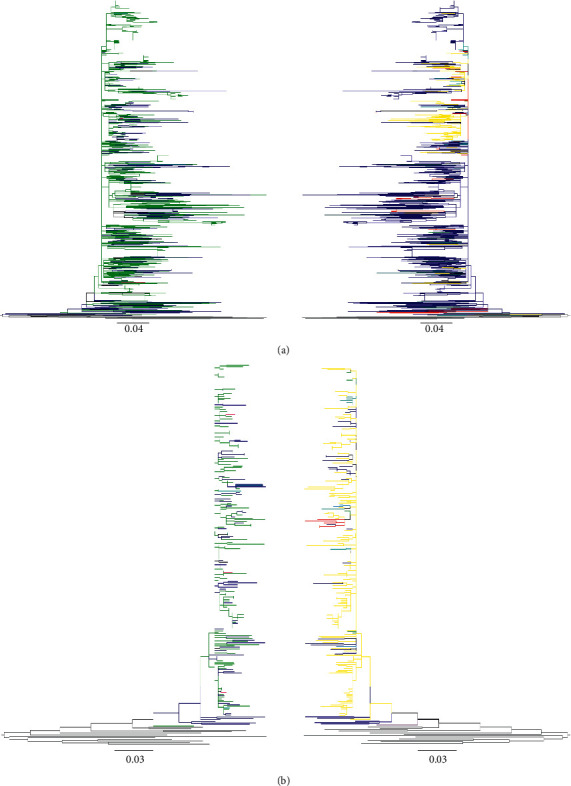
Analysis of HIV transmission routes in FSU countries: HIV-1 subtype A *env* (a) or *gag* (b) sequences from FSU countries were analysed for high-risk behavior. To analyse risk behavior in the context of location, the two versions of the same tree, with branches color-coded for the country of origin (left) or the associated risk group (right), are juxtaposed. Only sequences with recorded high-risk labels were included in this analysis. Risk behavior information for each sequence was obtained from HIV Los Alamos Database. For the tree on the right, the colors dark blue, teal, turquoise, pink, green, olive, red, neon, orange, and grey branches indicate, respectively, heterosexual, sexual transmission (unspecified type), MSM, mother-to-child, PWID, blood transfusion, homosexual, sex worker, bisexual, and reference sequences. For the tree on the left, the color key is the same as for Figure 4.

To analyse modes of transmission, high-risk data associated with HIV *env* and *gag* sequences were also analysed. Only sequences with recorded high-risk information were included in this analysis. Both the *env* (Figure 5(a)) and *gag* (Figure 5(b)) trees showed a predominance of PWID-associated sequences, followed by those linked with heterosexual transmission. Results represented here showed intermixing of PWID and heterosexual routes of transmission, indicating the possibility of bridging of the initial PWID-epidemic into heterosexual populations.

### 3.4. Bayesian Analysis

As summarized in Table 5, our Bayesian phylogenetic analysis showed substitution rate for NS5B HCV 1b and HCV 3a to be 6.8 (5.8–7.9) × 10^−3^ and 1.1 (0.7–1.4) × 10^−3^ substitutions per site per year, respectively, with a SkyGrid coalescent model, whereas the estimated tMRCAs for HCV 1b and HCV 3a were 1977 (95% highest posterior density, HPD: 1962–1985) and 1965 (95% HPD: 1941–1978), respectively. The estimated evolutionary rates of HIV *env* and HIV *gag* were 3.5 (2.5–4.0) × 10^−2^ and 13 (8.8–17) × 10^−3^ substitutions per site per year, respectively. The likely years of origins of HIV *env* and *gag* were estimated to be 1992 (95% HPD: 1990–1993) and 1996 (95% HPD: 1995–1997), respectively.

**Figure 6:**
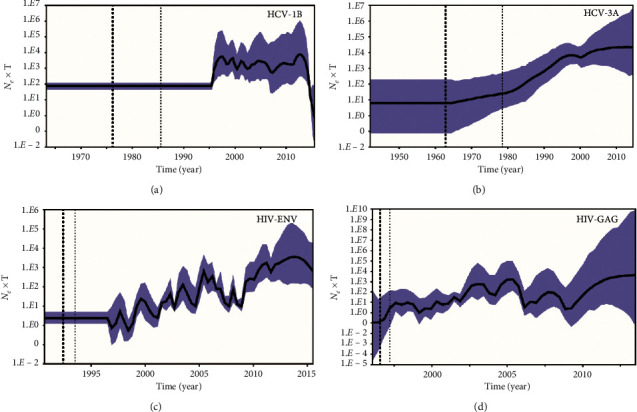
Population dynamics of the expansion of HCV genotypes 1B and 3A (top left and right panel, respectively) (NS5B) and HIV-1 subtype A (*env* and *gag*: bottom left and right panel, respectively). The population dynamics was analysed by Bayesian MCMC inference method with a SkyGrid demographic reconstruction model in BEAST. The vertical axis shows the effective number of infections (*N*_*e*_) multiplied by mean viral generation time (т). The solid line and shaded region represent the median and 95% credibility interval of *N*_*e*_т through time.

We further investigated the past population dynamics of HCV and HIV using a Bayesian skyline plot, which depicts the changes in effective population size over time. The effective population size of HCV 1b was observed to be fluctuating and slowly rising from 1998 to 2011. The change in effective population size for HCV 3a rose slowly and steadily starting in 1965, characterized by a phase of exponential growth (1981–1997), and the effective population size surged approximately 100-fold during the period. The epidemic history of HIV *env* and HIV *gag* seems to have experienced complex dynamics, characterized by two phases of wave-like fluctuating growth, during 1997–2005 and 2009–2014 for HIV *env*, and during 1995–2005 and 2009–2015 for HIV *gag* (Figure 6).

## 4. Discussion

In this study, we show evidence of transmission of two blood-borne viruses, HCV and HIV, within the FSU countries. According to our analysis, common routes of transmission appear to be injection drug use and heterosexual sex, with migrant mobility facilitating the cross-border spread of infection.

There have been reports of high prevalence of blood-borne infections in the FSU region. Within FSU countries, Russia has the highest rate of HCV genotype 1b (50%), followed by HCV 3a (45%) [13]. These two HCV genotypes also appear to be predominant in other FSU countries; Uzbekistan (1b, 64.2%; 3a, 25%), Azerbaijan (1b, 71.1%; 3a, 17%), Tajikistan (1b, 84.6%; 3a, 7.6%), Lithuania (1b, 54.3%; 3a, 23.9%), Estonia (1b, 71%; 3a, 24%), and Georgia (1b, 62%; 3a, 27%) [14–19].

In our phylogenetic tree constructed with HCV 1b sequences (Figure 1), a preponderance of Russian sequences was seen—a reflection of a higher representation of sequences from this country in the Los Alamos database. Russian sequences were intermixed with clusters from most other FSU countries, with a relatively higher proportion from Lithuania and Estonia. This might reflect a higher transmigration between Russia, Lithuania, and Estonia, due to their close geographic proximity. Conversely, in clusters C, D, and F, sequences from Azerbaijan, Uzbekistan, and Tajikistan were seen intermixed with those from Russia, indicating that Russia might have served as a hub for transmission across these countries. Phylogenetic tree constructed with HCV 3a NS5B sequences (Figure 2) showed an overall agreement with the 1b tree, showing trends of possible transmission within Russia, Lithuania, Uzbekistan, Estonia, and Azerbaijan.

Such phylogenetic patterns may be supported by historical and socioeconomic factors that came into play after the disintegration of the Soviet Union. It has been established that the first nationalism-related conflicts between Armenia and Azerbaijan (1987–1991), followed by Uzbekistan conflict (1989), and civil wars in Georgia (1991–1993) and Tajikistan (1992–1997) led to mass migration from these countries to Russia [1]. Sequences from these countries that show intermixing with those from Russia also date to roughly the same time (Tables 1 and 2). Our Bayesian analysis predicts origin of the HCV epidemics in FSU countries to be as early as 1965, for HCV3a, and 1977, for genotype 1b (Table 5 and Figure 6), indicating that these HCV genotypes were possibly circulating in the region before the conflicts started. It is possible that HCV was transmitted during this time through injection use, leading to a rise in transmission in the mid-1990s, as suggested by our Skyline plot analysis (Figure 6). In addition, significant reduction of employment opportunities and a dramatic decline in wages in Tajikistan and Georgia also facilitated a high flow of migrant labor into Russia. Further movement involved repatriates and internally displaced people of Russian-speaking origin into Russia owing to a resurgence of nationalism as a result of implementation of national language policies that discouraged use of Russian. It is thought that the reported numbers of migrants in this region is much lower than the actual numbers, since visa-free movement across borders does not account for illegally entering migrant workers [1].

In the initial stages of HIV outbreak in central Asian region, most people infected with HIV acquired a homogenic variant of subtype A, known as A_FSU_ (A6). This was thought to be a large-scale founder effect, resulting from transmigration within FSU countries [20]. The first HIV epidemic in FSU was recorded in the mid-1990s in Ukraine. A year later, outbreaks were detected in the European part of Russia, thereafter reaching other parts of Russia, Central Asia, and Eastern Europe.

The HIV epidemic expanded rapidly after the collapse of the USSR. As noted previously, political transitions led to the socioeconomic decline of newly formed independent counties. Consequently, large-scale labor migration to Russia from other countries of FSU provided epidemiological bridging of HIV transmission between Russia and the rest of FSU countries [20]. The database of international migration demonstrated a significant increase in migration between 1991 and 2000 within the FSU. The number of migrants from Central Asia, Caucasus, and Eastern Europe to Russia was highest from 1992 to 1996 [21], the same period during which our Skyline plot shows a gradual rise in the infections (Figure 6). Additionally, our phylogenetic trees revealed a dense intermixing of Russian sequences with other countries, dating around the same time (Figures 3 and 4 and Tables 3 and 4), which might suggest a link to migrant-associated HIV transmission. The HIV epidemic in FSU is considered to have initiated by transmissions through sexual contact and injection drug use [20]. Our results of phylogenetic tree in the context of high-risk behavior (Figures 5(a) and 5(b)) support that fact, showing the branches close to the ancestral node associated with heterosexual mode of transmission, with sequences represented from Uzbekistan (1999), Ukraine (1993), and Russia (1994).

As shown in previous reports, the initial HIV outbreak in FSU countries occurred in the mid-1990s in the city of Odessa, Ukraine, and spread to other countries of FSU [22]. Our phylogenetic tree also shows the ancestral node for the HIV subtype A epidemic originating from Ukrainian sequences deposited in 1993 (Figures 3 and 4), whereas the Bayesian analysis also predicts the origins of the epidemic to be around the same time (Table 1 and Figure 6). These results might indicate that Odessa had a favourable location for an explosive spread of HIV among PWID, as the city is situated on the Black Sea shore and was a major seaport and transportation hub during that period [20]. In the first postcollapse decade, there was an increase in injection drug use in the FSU [23], linked to the rise of drug production in Afghanistan during the mid-1990s and a shift in trafficking to the “Northern route,” which runs through Central Asia to Russia and South Caucasus to Eastern Europe [24, 25]. This increase in drug trafficking may have played a role in the early spread of HIV among PWID communities. Our phylogenetic tree also shows a high number of transmissions associated with drug use, later shifting the mode of transmission toward heterosexual sex. Furthermore, our phylogenetic analysis shows heavy intermixing of Armenian PWID sequences from the South Caucasian region, which is within “Northern route” of drug trafficking, with other Eastern European sequences. During the period of 1989–2001, another important scenario that may have facilitated transmission of HIV and HCV was the displacement of a population of about one million Armenians in aftermath of the 1988 earthquake, coupled with deteriorating socioeconomic situation in the 1990s. These displaced labor populations immigrated mainly to the Eastern European countries of Ukraine, Belarus, and Eastern part of Russia [26].

## 5. Conclusion

Our analyses highlight the main routes of transmission for two important blood-borne viruses in FSU countries. This information may be exploited to refine Public Health policies to better manage the infected populations and prevent further spread of the infections. Although this study was limited by the availability of the number of sequences from FSU countries in the open-access databases, the results highlight the importance of focusing the harm reduction efforts on communities where blood-borne infections might be currently spreading, namely, persons who inject drugs, heterosexual populations, migrant workers, and cross-border travelers.

## Figures and Tables

**Table 1:** Cluster analysis of HCV 1b *NS5B* tree in Figure 1 showing distribution of sequences among FSU countries.

Cluster	Number of sequences	Number of countries	Countries	Years
A	53	5	Uzbekistan, Russia, Estonia, Azerbaijan, Tajikistan	1997–2014
B	37	7	Uzbekistan, Russia, Estonia, Azerbaijan, Tajikistan, Georgia, Lithuania	1997–2014
C	18	4	Uzbekistan, Russia, Azerbaijan, Lithuania	1999–2014
D	23	6	Uzbekistan, Russia, Estonia, Azerbaijan, Tajikistan, Lithuania	1997–2014
E	133	6	Uzbekistan, Russia, Estonia, Azerbaijan, Tajikistan, Lithuania	1995–2014
F	33	5	Uzbekistan, Russia, Estonia, Tajikistan, Lithuania	1997–2006
G	30	5	Uzbekistan, Russia, Estonia, Azerbaijan, Lithuania	1998–2014
H	31	4	Russia, Estonia, Tajikistan, Lithuania	1998–2015
I	39	6	Uzbekistan, Russia, Estonia, Azerbaijan, Tajikistan, Lithuania	1995–2014
J	32	5	Uzbekistan, Russia, Estonia, Tajikistan, Lithuania	1997–2014

**Table 2:** Cluster analysis of HCV 3a *NS5B* tree in Figure 2 showing distribution of sequences among FSU countries.

Cluster	Number of sequences	Number of countries	Countries	Years
A	44	6	Uzbekistan, Russia, Estonia, Lithuania, Azerbaijan, Georgia	1998–2012
B	11	3	Lithuania, Russia, Azerbaijan	1998–2011
C	11	4	Uzbekistan, Russia, Lithuania, Estonia	2001–2006
D	18	3	Russia, Estonia, Azerbaijan	1998–2009
E	23	3	Uzbekistan, Russia, Estonia	1998–2009
F	80	6	Uzbekistan, Russia, Estonia, Lithuania, Azerbaijan, Tajikistan	1999–2014
G	33	4	Uzbekistan, Russia, Azerbaijan, Estonia	1999–2008

**Table 3:** Cluster analysis of HIV *env* tree in Figure 3 showing distribution of sequences among FSU countries.

Cluster	Number of sequences	Number of countries	Countries	Years
A	76	5	Russia, Uzbekistan, Estonia, Kyrgyzstan, Armenia	1999–2012
B	53	4	Russia, Latvia, Uzbekistan, Estonia	1999–2015
C	25	2	Russia, Latvia	2001–2012
D	83	6	Russia, Latvia, Uzbekistan, Belarus, Ukraine, Moldova	1996–2012
E	13	3	Russia, Uzbekistan, Ukraine	1995–2012
F	111	4	Russia, Latvia, Armenia, Estonia	1998–2013
G	143	5	Russia, Latvia, Uzbekistan, Armenia, Ukraine	1993–2015
H	8	4	Russia, Latvia, Uzbekistan, Armenia	1999–2009
I	17	5	Russia, Latvia, Uzbekistan, Moldova, Armenia	1997–2011
J	55	4	Russia, Latvia, Armenia, Belarus	2004–2015
K	130	4	Russia, Latvia, Armenia, Ukraine	2001–2015
L	16	2	Russia, Latvia	2001–2015
M	84	5	Russia, Latvia, Armenia, Ukraine, Georgia	1999–2015
N	16	3	Russia, Armenia, Ukraine	2009–2015
O	6	1	Ukraine	1993

**Table 4:** Cluster analysis of HIV *gag* tree in Figure 4 showing distribution of sequences among FSU countries.

Cluster	Number of sequences	Number of countries	Countries	Years
A	10	1	Latvia	2001–2005
B	13	3	Russia, Latvia, Ukraine	2000–2003
C	9	4	Russia, Latvia, Kazakhstan, Uzbekistan	2002–2005
D	7	3	Russia, Latvia, Ukraine	2001–2011
E	6	4	Russia, Latvia, Ukraine, Belarus	1997–2005
F	17	1	Latvia	1998–2002
G	33	4	Russia, Latvia, Ukraine, Belarus	1997–2006
H	13	1	Latvia	2001–2003
I	12	2	Latvia, Russia	2001–2005
J	20	3	Russia, Latvia, Ukraine	1998–2008
K	31	1	Latvia	2001–2005
L	7	3	Russia, Kazakhstan, Latvia	2002–2006

**Table 5:** tMRCA and Substitution rate of HCV and HIV.

	tMRCA (median and 95% HPD interval)	Substitution/site/year (median and 95% HPD interval)
HCV 1B	37.7 (30.0, 52.6)	6.8*E* – 3 (5.8*E* –3, 7.9*E* – 3)
HCV 3A	49.3 (36.0, 73.1)	1.1*E* – 3 (7.0*E* – 4, 1.4*E* – 3)
HIV *ENV*	22.8 (22.0, 24.8)	3.5*E* – 2 (2.5*E* – 2, 4.0*E* – 2)
HIV *GAG*	16.9 (16.3, 17.5)	1.3*E* – 2 (8.8*E* – 3, 1.7*E* – 2)

## Data Availability

The data used to support the findings of this study are available from the corresponding author.

## Supplementary Materials

Supplementary Materials Supplementary Figure 1: numbers of HIV and HCV sequences available in Los Alamos Database are shown for each country. HCV Genotype 1b NS5B, HCV Genotype 3a NS5B, HIV Subtype A env, and HIV Subtype A gag are color-coded in boxes as yellow, green, light blue, and dark blue, respectively.
