# Supplementary material for: Transmission of HIV and HCV within Former Soviet Union Countries
Source: Can J Gastroenterol Hepatol. 2020 Jul 15;2020:9701920. doi: 10.1155/2020/9701920 (PMC7378597; doi:10.1155/2020/9701920)
Supplement: Supplementary Materials — Supplementary Figure 1: numbers of HIV and HCV sequences available in Los Alamos Database are shown for each country. HCV Genotype 1b NS5B, HCV Genotype 3a NS5B, HIV Subtype A env, and HIV Subtype A gag are color-coded in boxes as yellow, green, light blue, and dark blue, respectively. [file 9701920.f1.pptx]

## Slide 1
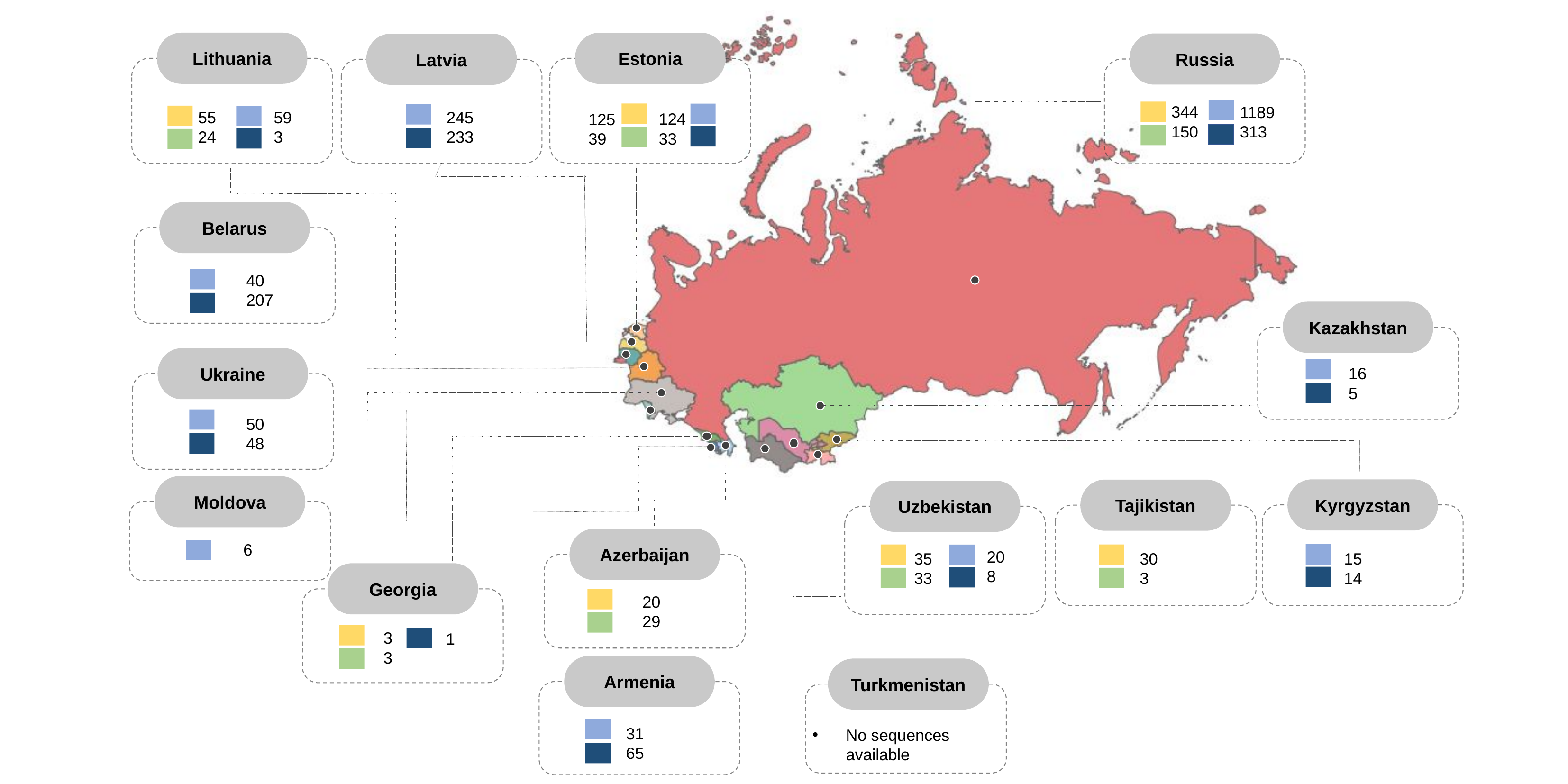

Lithuania
55
24
59
3
Estonia
125
39
Russia
344
150
1189
313
Latvia
245
233
124
33
Belarus
40
207
Kazakhstan
16
5
Ukraine
50
48
Azerbaijan
20
29
Moldova
6
Kyrgyzstan
15
14
Tajikistan
30
3
Uzbekistan
35
33
20
8
Georgia
3
3
1
Armenia
31
65
Turkmenistan
No sequences available
